# Cytotoxic and Enzyme Inhibitory Potential of Two *Potentilla* species (*P. speciosa* L. and *P. reptans* Willd.) and Their Chemical Composition

**DOI:** 10.3389/fphar.2017.00290

**Published:** 2017-05-23

**Authors:** Sengul Uysal, Gokhan Zengin, Marcello Locatelli, Mir B. Bahadori, Andrei Mocan, Giuseppe Bellagamba, Elisa De Luca, Adriano Mollica, Abdurrahman Aktumsek

**Affiliations:** ^1^Department of Biology, Science Faculty, Selcuk University, CampusKonya, Turkey; ^2^Department of Pharmacy, University “G. d’Annunzio” Chieti-PescaraChieti, Italy; ^3^Interuniversity Consortium of Structural and Systems BiologyRome, Italy; ^4^Research Center for Pharmaceutical Nanotechnology, Tabriz University of Medical SciencesTabriz, Iran; ^5^Department of Pharmaceutical Botany, “Iuliu Hatieganu” University of Medicine and PharmacyCluj-Napoca, Romania

**Keywords:** *Potentilla reptans*, *P. speciosa*, antioxidant activity, enzyme inhibitory activity, cytotoxic

## Abstract

In this work, the biological and chemical fingerprints of three extracts (ethyl acetate, methanol, and water) from two *Potentilla* species (*Potentilla reptans* and *P. speciosa*) were investigated. Antioxidant, enzyme inhibitory, and cytotoxic activities were performed for the biological fingerprint. For the chemical characterization, total bioactive components, and individual phenolic components were determined using photometric and HPLC methods, respectively. The main identified phenolic compounds in these extracts were rutin and catechin. Methanol and water extracts contained the highest total phenolic and flavonoid content. The results of antioxidant assays showed that methanol and water extracts displayed higher antioxidant activity compared to the ethyl acetate extract. Generally, methanol and water extracts exhibited higher biological activities correlated with higher levels the bioactive components. For *P. speciosa*, the methanol extract exhibited the highest enzyme inhibitory activity (except BChE inhibitory activity). *P. reptans* exhibited also high antiproliferative activity against MCF-7 cells whilst *P. speciosa* had weak to moderate activity against both of A549 and MCF-7 cell lines. The results suggest that *Potentilla* species could be potential candidates for developing new phyto-pharmaceuticals and functional ingredients.

## Introduction

Natural products and functional-food ingredients gained interest due to their valuable biological effects including antioxidant, anticancer or antimicrobial. Currently, studies on natural products and medicinal plants are one of the most important subjects in pharmaceutical area (Sut et al., 2016). In these studies, many plants or plant-derived products are suggested as potential agents for designing new pharmaceuticals or food ingredients (Raskin et al., 2002). However, there is still limited knowledge about chemical and biological profiles of many wild plant species used as folk remedies in traditional medicine.

Alzheimer’s disease (AD) and Diabetes mellitus (DM) are considered major global health problems in the 21st century (Waltenberger et al., 2016). Today, the prevalence of AD and DM is rising and is estimated to increase significantly over the next two decades. Consequently, many therapeutic strategies are developed for these health problems, and the key enzyme inhibitory theory is one of the most accepted approaches. Acetylcholinesterase (AChE) and butyrylcholinesterase (BChE) are the enzymes into synaptic cleft that terminates the cholinergic signal transfer, and which are considered targets for the treatment of AD. α-amylase and α-glucosidase are main enzymes involved in the catabolism of carbohydrates and they are of vital importance for decreasing post-prandial blood glucose level. Tyrosinase is a key enzyme in melanin biosynthesis and thus the inhibition of this enzyme is associated with the prevention of skin disorders (SD) (Schelterns and Feldman, 2003). Several synthetic and natural inhibitors (galantamine and tacrine for AD; acarbose and voglibose for DM; kojic acid for SD) were developed for the management of these diseases by drug industry. However, many researches have reported that synthetic inhibitors have unfavorable effects such as nausea and diarrhea (Nouri et al., 2014; Anantharaman et al., 2016; Bekir et al., 2016). Due to these adverse effects, there is an increasing search for inhibitors derived from natural products (non-toxic and effective) against key enzymes related with these diseases (Day, 1998; Qin et al., 2013).

The genus *Potentilla* belongs to the Rosaceae family and is represented by about 500 species around the world (Tomczyk and Latté, 2009). Also, the genus comprises about 53 species in Turkish Flora (Pesmen, 1972). *Potentilla* species have been used as traditional medicine for the treatment of various diseases. For example, *P. fulgens* Lodd. is used for the treatment of DM, cancer, stomach disorders, cough, and as wound healing (Syiem et al., 2002; Rosangkima and Prasad, 2004; Jaitak et al., 2010; Roy et al., 2010). *P. mooniana* Wight. is used to treat gastric problems and mouth ulcers (Ahmed and Borthakur, 2005; Selvam, 2008). Also, *P. fruticosa* L. has several medicinal properties including strengthening the stomach and the spleen, promoting metabolism, and it is widely used as a tea (Miliauskas et al., 2004; Li et al., 2007; Liu et al., 2016). Furthermore, *P. atrosanguinea* Lodd. has been used for wound healing, treating diarrhea, and influenza. Owing their potential uses in different purposes, several studies focused on the biological effects and chemical profile of the genus *Potentilla* (Tomczyk et al., 2010; Tomovic et al., 2015; Uysal and Aktumsek, 2015). However, to the best of our knowledge, the biological and chemical fingerprints of *Potentilla reptans* Willd and *P. speciosa* L. have not yet been reported. Thus, the main purpose of present study is to evaluate biological (antioxidant capacity, enzyme inhibitory, and cytotoxic activities) and chemical (total bioactive components and individual phenolic compositions) fingerprints of *P. reptans* and *P. speciosa*. The obtained results will provide new insights on the members of this genus for potential phyto-pharmaceuticals and nutraceuticals development.

## Materials and Methods

### Plant Materials

Taxonomic identification of the plant material was kindly confirmed by senior taxonomist Dr. Murad Aydın SANDA, from Department of Field Crops, Agriculture Faculty, Igdir University, Igdir, Turkey. Voucher specimens have been deposited at the Herbarium of the Department of Biology, Selcuk University, Konya, Turkey. Localities and collection periods of *Potentilla* species are as following:

*P. reptans*: Selcuk University, Alaaddin Keykubad Campus, Konya, Turkey, June 2015, (Voucher Number: GZ-1532).

*P. speciosa*: Nigde, Camardi, Mazmili Mountain, Turkey, July 2015 (Voucher Number: GZ-1560).

### Preparation of the Extracts

Aerial parts plant materials were air-dried at room temperature. The dried plant materials were ground to a fine powder using a laboratory mill. The powdered plant samples (10 g) were extracted with 250 mL of solvent (ethyl acetate, methanol) using a Soxhlet apparatus for 6–8 h. Extracts were then filtered and concentrated under vacuum at 40°C by using a rotary evaporator. To obtain water extracts, powdered *P. reptans* and *P. speciosa* aerial parts (15 g) were boiled with 250 mL of distilled water for 30 min. The water extracts were then filtered and lyophilized [–80°C, 48 h]. Extracts were kept at 4°C (±1°C) in dark until further analysis. Abbreviation for these extracts are; Pr-EA (*Potentilla reptans* ethyl acetate), Pr-Met (*P. reptans* methanol), Pr-Wat (*P. reptans* water), Ps-EA (*Potentilla speciosa* ethyl acetate), Ps-Met (*P. speciosa* methanol), Ps-Wat (*P. speciosa* water).

### Total Phenolics, Flavonoid, Saponins, Triterpenoids, and Phenolic Composition

The total phenolic content was determined by employing the methods given in the literature (Slinkard and Singleton, 1977) with some modification. Sample solution (1 mg/mL; 0.25 mL) was mixed with diluted Folin–Ciocalteu reagent (1 mL, 1:9, v/v) and shaken vigorously. After 3 min, Na_2_CO_3_ solution (0.75 mL, 1%) was added and the sample absorbance was read at 760 nm after a 2 h incubation at room temperature. The total phenolic content was expressed as milligrams of gallic acid equivalents (mg GAE/g extract) (Vlase et al., 2014).

The total flavonoids content was determined using AlCl_3_ method (Zengin et al., 2014). Briefly, sample solution (1 mg/mL; 1 mL) was mixed with the same volume of aluminum trichloride (2%) in methanol. Similarly, a blank was prepared by adding sample solution (1 mL) to methanol (1 mL) without AlCl_3_. The sample and blank absorbances were read at 415 nm after a 10 min incubation at room temperature. The absorbance of the blank was subtracted from that of the sample. Rutin was used as a reference standard and the total flavonoid content was expressed as milligrams of rutin equivalents (mg RE/g extract) (Mocan et al., 2015).

The total saponins content of the extract was determined by the vanillin-sulfuric acid method (Aktumsek et al., 2013). Sample solution (1 mg/mL; 0.25 mL) was mixed with vanillin (0.25 mL, 8%) and sulfuric acid (2 mL, 72%). The mixture was incubated for 10 min at 60°C. Then the mixture was cooled for another 15 min, followed by the sample absorbance measurement at 538 nm. The total saponin content was expressed as milligrams of quillaja equivalents (mg QAE/g extract).

The total triterpenoids content of the extracts was determined according to Zhang et al. (2010) method with some modifications. Briefly, sample solution (1 mg/mL; 500 μL) was mixed with the vanillin–glacial acetic acid (5%, w/v, 0.5 mL) and 1 mL of perchloric acid. The mixture was incubated at 60°C for 10 min, cooled in an ice water bath for 15 min and then 5 mL glacial acetic acid was added and mixed well. After 6 min, the absorbance was read at 538 nm. Oleanolic acid was used as a reference standard and the content of total triterpenoids was expressed as oleanolic acid equivalents (mg OAE/g extract) through a calibration curve with oleanolic acid.

HPLC-PDA analyses were performed on a Waters liquid chromatograph equipped with a model 600 solvent pump and a 2996 photodiode array detector, and Empower v.2 Software (Waters Spa, Milford, MA, United States) was used for acquisition of data. A C18 reversed-phase packing column (Prodigy ODS (3), 4.6 × 150 mm, 5 μm; Phemomenex, Torrance, CA, United States) was used for the separation and the column was thermostated at 30 ± 1°C using a Jetstream2 Plus column oven. The injection volume was 20 μL. The mobile phase was directly on-line degassed by using Biotech DEGASi, mod. Compact (LabService, Anzola dell’Emilia, Italy). Gradient elution was performed using the mobile phase water-acetonitrile (93:7, v/v, 3% acetic acid) (Zengin et al., 2016). The UV/Vis acquisition wavelength was set in the range of 200–500 nm. The quantitative analyses were achieved at maximum wavelength for each compound.

### Biological Activities Evaluation

Antioxidant (DPPH and ABTS radical scavenging, reducing power (CUPRAC and FRAP), phosphomolybdenum, and metal chelating (ferrozine method)) and enzyme inhibitory activities [cholinesterase (ChE) Elmann’s method], tyrosinase (dopachrome method), α-amylase (iodine/potassium iodide method), and α -glucosidase (chromogenic PNPG method)) were determined using the methods previously described by Zengin et al. (2014) and Dezsi et al. (2015).

For the DPPH (1,1-diphenyl-2-picrylhydrazyl) radical scavenging assay: Sample solution (1 mg/mL; 1 mL) was added to 4 mL of a 0.004% methanol solution of DPPH. The sample absorbance was read at 517 nm after a 30 min incubation at room temperature in the dark. DPPH radical scavenging activity was expressed as millimoles of trolox equivalents (mg TE/g extract).

For ABTS (2,2′-azino-bis(3-ethylbenzothiazoline) 6-sulfonic acid) radical scavenging assay: Briefly, ABTS+ was produced directly by reacting 7 mM ABTS solution with 2.45 mM potassium persulfate and allowing the mixture to stand for 12–16 in the dark at room temperature. Prior to beginning the assay, ABTS solution was diluted with methanol to an absorbance of 0.700 ± 0.02 at 734 nm. Sample solution (1 mg/mL; 1 mL) was added to ABTS solution (2 mL) and mixed. The sample absorbance was read at 734 nm after a 30 min incubation at room temperature. The ABTS radical scavenging activity was expressed as millimoles of trolox equivalents (mmol TE/g extract) (Mocan et al., 2016a).

For CUPRAC (cupric ion reducing activity) activity assay: Sample solution (1 mg/mL; 0.5 mL) was added to premixed reaction mixture containing CuCl_2_ (1 mL, 10 mM), neocuproine (1 mL, 7.5 mM) and NH_4_Ac buffer (1 mL, 1 M, pH 7.0). Similarly, a blank was prepared by adding sample solution (0.5 mL) to premixed reaction mixture (3 mL) without CuCl_2_. Then, the sample and blank absorbances were read at 450 nm after a 30 min incubation at room temperature. The absorbance of the blank was subtracted from that of the sample. CUPRAC activity was expressed as milligrams of trolox equivalents (mg TE/g extract).

For FRAP (ferric reducing antioxidant power) activity assay: Sample solution (1 mg/mL; 0.1 mL) was added to premixed FRAP reagent (2 mL) containing acetate buffer (0.3 M, pH 3.6), 2,4,6-tris(2-pyridyl)-*S*-triazine (TPTZ) (10 mM) in 40 mM HCl and ferric chloride (20 mM) in a ratio of 10:1:1 (v/v/v). Then, the sample absorbance was read at 593 nm after a 30 min incubation at room temperature. FRAP activity was expressed as milligrams of trolox equivalents (mg TE/g extract).

For phosphomolybdenum method: Sample solution (1 mg/mL; 0.3 mL) was combined with 3 mL of reagent solution (0.6 M sulfuric acid, 28 mM sodium phosphate and 4 mM ammonium molybdate). The sample absorbance was read at 695 nm after a 90 min incubation at 95°C. The total antioxidant capacity was expressed as millimoles of trolox equivalents (mmol TE/g extract) (Mocan et al., 2016c).

For metal chelating activity assay: Briefly, sample solution (1 mg/mL; 2 mL) was added to FeCl_2_ solution (0.05 mL, 2 mM). The reaction was initiated by the addition of 5 mM ferrozine (0.2 mL). Similarly, a blank was prepared by adding sample solution (2 mL) to FeCl_2_ solution (0.05 mL, 2 mM) and water (0.2 mL) without ferrozine. Then, the sample and blank absorbances were read at 562 nm after 10 min incubation at room temperature. The absorbance of the blank was sub-tracted from that of the sample. The metal chelating activity was expressed as milligrams of EDTA (disodium edetate) equivalents (mg EDTAE/g extract).

For ChE inhibitory activity assay: Sample solution (1 mg/mL; 50 μL) was mixed with DTNB (5,5-dithio-bis(2-nitrobenzoic) acid, Sigma, St. Louis, MO, United States) (125 μL) and AChE [acetylcholines-terase (Electric ell AChE, Type-VI-S, EC 3.1.1.7, Sigma)], or BChE [BChE (horse serum BChE, EC 3.1.1.8, Sigma)] solution (25 μL) in Tris–HCl buffer (pH 8.0) in a 96-well microplate and incubated for 15 min at 25°C. The reaction was then initiated with the addition of acetylthiocholine iodide (ATCI, Sigma) or butyrylthiocholine chloride (BTCl, Sigma) (25 μL). Similarly, a blank was prepared by adding sample solution to all reaction reagents without enzyme (AChE or BChE) solution. The sample and blank absorbances were read at 405 nm after 10 min incubation at 25°C. The absorbance of the blank was subtracted from that of the sample and the cholinesterase inhibitory activity was expressed as galanthamine equivalents (mgGALAE/g extract) (Mocan et al., 2016b).

For Tyrosinase inhibitory activity assay: Sample solution (1 mg/mL; 25 μL) was mixed with tyrosinase solution (40 μL, Sigma) and phosphate buffer (100 μL, pH 6.8) in a 96-well microplate and incubated for 15 min at 25°C. The reaction was then initiated with the addition of L-DOPA (40 μL, Sigma). Similarly, a blank was prepared by adding sample solution to all reaction reagents without enzyme (tyrosinase) solution. The sample and blank absorbances were read at 492 nm after a 10 min incubation at 25°C. The absorbance of the blank was subtracted from that of the sample and the tyrosinase inhibitory activity was expressed as kojic acid equivalents (mgKAE/g extract) (Mocan et al., 2017).

For α-amylase inhibitory activity assay: Sample solution (1 mg/mL; 25 μL) was mixed with α-amylase solution (ex-porcine pancreas, EC 3.2.1.1, Sigma) (50 μL) in phosphate buffer (pH 6.9 with 6 mM sodium chloride) in a 96-well microplate and incubated for 10 min at 37°C. After pre-incubation, the reaction was initiated with the addition of starch solution (50 μL, 0.05%). Similarly, a blank was prepared by adding sample solution to all reaction reagents without enzyme (α-amylase) solution. The reaction mixture was incubated 10 min at 37°C. The reaction was then stopped with the addition of HCl (25 μL, 1 M). This was followed by addition of the iodine-potassium iodide solution (100 μL). The sample and blank absorbances were read at 630 nm. The absorbance of the blank was subtracted from that of the sample and the α-amylase inhibitory activity was expressed as acarbose equivalents (mmol ACE/g extract) (Savran et al., 2016).

For α-glucosidase inhibitory activity assay: Sample solution (1 mg/mL; 50 μL) was mixed with glutathione (50 μL), α-glucosidase solution (from Saccharomyces cerevisiae, EC 3.2.1.20, Sigma) (50 μL) in phosphate buffer (pH 6.8) and PNPG (4-*N*-trophenyl-α-D-glucopyranoside, Sigma) (50 μL) in a 96-well microplate and incubated for 15 min at 37°C. Similarly, a blank was prepared by adding sample solution to all reaction reagents without enzyme (α-glucosidase) solution. The reaction was then stopped with the addition of sodium carbonate (50 μL, 0.2 M). The sample and blank absorbances were read at 400 nm. The absorbance of the blank was subtracted from that of the sample and the α-glucosidase inhibitory activity was expressed as acarbose equivalents (mmol ACE/g extract) (Llorent-Martínez et al., 2016).

All the assays were carried out in triplicate. The results are expressed as mean values and standard deviation (SD). The differences between the different extracts were analyzed using one-way analysis of variance (ANOVA) followed by Tukey’s honestly significant difference *post hoc* test with α = 0.05. This treatment was carried out using SPSS v. 14.0 program.

### Cell Viability Assay

Cell viability assay was performed for the extracts of the two *Potentilla* species (Farimani et al., 2015). Human alveolar lung epithelial carcinoma (A549) and human breast adenocarcinoma (MCF-7) cells were cultured in 75 cm^2^ flasks containing RPMI 1640 medium supplemented, 10% FBS (fetal bovine serum) and antibiotics (100 mg/mL penicillin/streptomycin). Cells were grown in an atmosphere of 5% CO_2_ at 37°C (±1°C) with 95% humidity. The antiproliferative activities of *Potentilla* extracts were determined against A549 and MCF-7 cells using the MTT assay. Cells were seeded in 96-well plates (2 × 10^4^ cells per well) and maintained at 37°C (±1°C) with 5% CO_2_ atmosphere for 24 h before test extracts were added as DMSO solutions. Stock solutions were prepared by dissolving *Potentilla* extracts in DMSO (100 mg/mL) to reach a final DMSO concentration of 0.1%. Equal volume of DMSO (0.1%) was added into untreated wells. After incubation (for 24, 48, and 72 h), 50 μL of MTT solution (2 mg/mL in phosphate buffer saline) was added to each well. Afterwards, the plates were incubated for additional 4 h. DMSO was used for formazan solubilization and its UV absorbance was measured at 570 nm. Doxorubicin was used as the positive control. The percentage of cytotoxicity was calculated based on the comparison with untreated cells. All of the experiments were carried out in quadruplicate and the IC_50_ values were expressed as average ± SD. Statistical comparisons were estimated by one-way ANOVA followed by Duncan’s *post hoc* test for multiple comparisons with control. Statistical analyses were performed using SPSS 16.0 software. A value of *p* < 0.05 was considered to indicate statistical significance.

## Results and Discussion

### Extraction Yield and Identification of Phenolic Compounds

*Potentilla reptans* and *P. speciosa* were extracted using different solvents (ethyl acetate, methanol, and water) and extraction yields of samples are shown in **Table 1**. The solvents used for extraction play a significant role on the extraction yield. The extraction yields increased in the following order: methanol > water > ethyl acetate. The highest extraction yield was for Pr-Met (20.61%).

**Table 1:** Extraction yield and total bioactive components of different solvent extracts obtained from of *Potentilla reptans* and *P. speciosa^∗^.*

Assays	*Potentilla reptans*			*Potentilla speciosa*		
		
	Ethyl acetate	Methanol	Water	Ethyl acetate	Methanol	Water
Extraction yield (%)	3.29	20.61	13.95	3.21	8.60	4.07
Total phenolics (mg GAEs/g extract)^a^	42.13 ± 0.36c	111.68 ± 0.65b	135.73 ± 3.94a	24.98 ± 0.50c	102.58 ± 2.27b	138.45 ± 1.54a
Total flavonoids (mg REs/g extract)^b^	25.10 ± 0.38c	37.95 ± 0.70a	30.56 ± 0.11b	9.34 ± 0.13c	29.83 ± 0.21a	16.30 ± 0.24b
Total saponins (mg QEs/g extract)^c^	506.81 ± 33.84a	459.93 ± 30.68b	268.96 ± 18.09c	347.56 ± 71.26c	928.05 ± 56.65a	575.58 ± 22.41b
Total triterpenoids (mg OAE/g extract)^d^	4.21 ± 0.12a	2.39 ± 0.07b	0.64 ± 0.01c	2.20 ± 0.13b	5.17 ± 0.03a	2.04 ± 0.07b


*^a^GAEs, gallic acid equivalents; ^b^REs, rutin equivalents; ^c^QEs, quillaja equivalents; ^d^OAEs, oleanolic acid equivalents. ^∗^Values expressed are means ± SD; Data marked with different letters within the same row indicate statistically significant differences for each sample (*p* < 0.05).*

**FIGURE 1:**
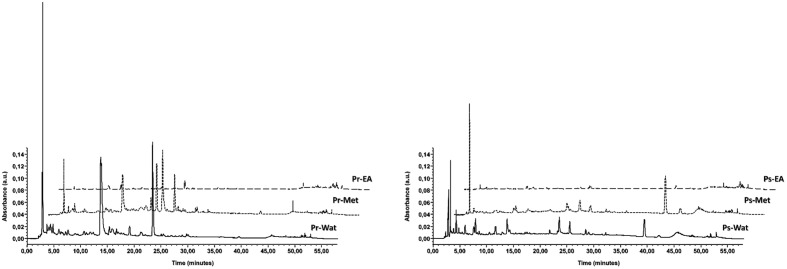
**HPLC-PDA analyses of the three different extracts (ethyl acetate, methanol, and water) for the two *Potentilla* species; chromatographic profiles are reported at 280 nm**.

**FIGURE 2:**
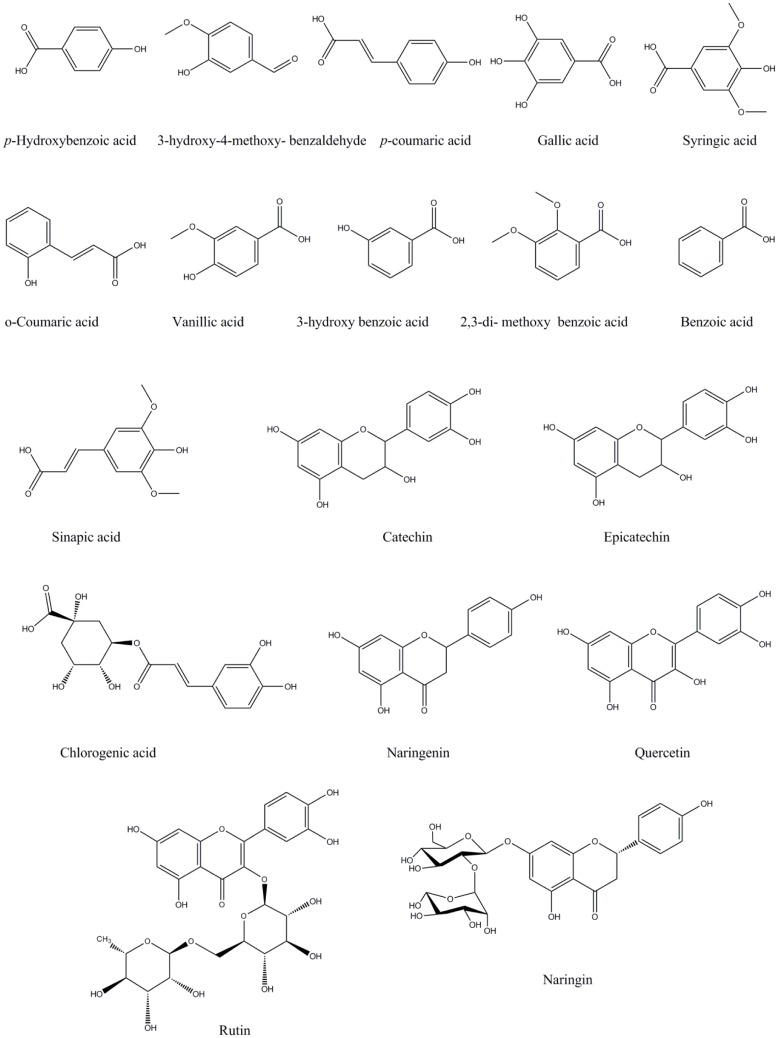
**Phenolic compounds identified in *Potentilla* species**.

To understand the relationship between antioxidant capacity and phenolic components, the phenolic components of two *Potentilla* species were determined by using HPLC-PDA (**Figure 1**). As shown in **Table 2**, two major compounds in all samples were identified as catechin (0.52–7.30 mg/g extract) and rutin (8.09–51.51 mg/g extract). Chemical structures of all identified phenolic compounds in *Potentilla* extracts are presented in **Figure 2**. These results are supported by the findings of Wang et al. (2013) who reported that catechin, rutin, and ellagic acid were the most abundant compounds in *P. parvifolia* Fisch. ex Lehm. Furthermore, the aerial parts of *P. fruticosa* have been found to contain these phenolics (particularly elagic acid, catechins, and flavonols) (Fedoseeva, 1979). Catechin has been reported to be present in roots and rhizomes of *P. erecta* L., *P. anserina* L., *P. alba* L., and *P. viscosa* Donn ex Lehm. (Gritsenko and Smik, 1977; Zhang et al., 1988; Vennat et al., 1992; Kombal and Glasl, 1995). Furthermore, catechin has been isolated from aerial parts of some *Potentilla* species such as *P. erecta*, *P. fruticosa*, and *P. fragarioides* L. (Goncharov et al., 1989; Kombal and Glasl, 1995; Choi et al., 1998; Miliauskas et al., 2004). According to the study of Tomczyk and Latté (2009), the dominant components in aerial parts of *Potentilla* species were flavonoids. A number of flavonoids (such as apigenin, kaempferol, quercetin, naringenin) have been identified from some *Potentilla* species (*P. viscosa*, *P. multifidi* L., *P. discolor* Bunge, and *P. erecta*) (Liu et al., 1984; Goncharov et al., 1989; Xue et al., 2005; Shen et al., 2006). Biological properties of rutin have been also reported including antibacterial, antitumor, anti-inflammatory (Calabro et al., 2005), antiallergic (Zwirtes de Oliveira et al., 2006), anticarcinogenic (Webster et al., 1996), and antioxidant (Yang et al., 2008). On the basis of these considerations, rutin and catechin could play an important role in the biological effects of the investigated *Potentilla* extracts. The other phenolic compounds were observed in minor amounts. Ferulic and cinnamic acids were not detected in any sample. Nonetheless, tannins and triterpenoids are also known as significant biologically active components of the genus *Potentilla* (Xue et al., 2005; Liu et al., 2006; Tomczyk and Latté, 2009).

**Table 2:** Phenolic compounds of *P. reptans* and *P. speciosa* (mg/g extract)*^∗^.*

No	Compounds	Retention Time (min)	Wavelength (nm)	*Potentilla reptans*			*Potentilla speciosa*		
					
				Ethyl acetate	Methanol	Water	Ethyl acetate	Methanol	Water
1	Gallic acid	4.40 ± 0.06	271	0.10 ± 0.03b	0.02 ± 0.01c	0.80 ± 0.13a	0.10 ± 0.04b	0.15 ± 0.04b	1.49 ± 0.45a
2	Catechin	11.9 ± 0.27	278	4.89 ± 0.98a	nd	0.52 ± 0.17b	2.28 ± 0.85c	5.53 ± 1.02b	7.30 ± 1.28a
3	Chlorogenic acid	12.7 ± 0.23	324	0.03 ± 0.01b	nd	0.16 ± 0.07a	nd	nd	0.21 ± 0.07
4	*p*-OH benzoic acid	13.2 ± 0.20	256	nd	0.02 ± 0.01a	0.03 ± 0.01a	0.34 ± 0.09a	0.27 ± 0.07b	0.33 ± 0.08a
5	Vanillic acid	15.7 ± 0.19	260	0.16 ± 0.05b	0.67 ± 0.06a	0.09 ± 0.01c	0.19 ± 0.07a	0.17 ± 0.05a	0.20 ± 0.09a
6	Epicatechin	16.4 ± 0.23	278	0.07 ± 0.02b	0.70 ± 0.11a	nd	0.15 ± 0.03b	nd	0.34 ± 0.10a
7	Syringic acid	16.7 ± 0.17	274	nd	0.06 ± 0.01b	1.90 ± 0.23a	nd	nd	nd
8	3-OH benzoic acid	16.9 ± 0.21	295	nd	nd	0.47 ± 0.18	nd	nd	0.16 ± 0.09
9	3-OH-4-MeO benzaldehyde	20.4 ± 0.18	275	nd	5.63 ± 1.07	nd	0.03 ± 0.01b	0.83 ± 0.15a	nd
10	*p*-coumaric acid	22.0 ± 0.19	309	0.06 ± 0.01a	0.01 ± 0.005b	nd	0.15 ± 0.07c	0.19 ± 0.06b	0.29 ± 0.11a
11	Rutin	24.0 ± 0.14	256	8.09 ± 1.01c	21.80 ± 3.58b	51.51 ± 5.19a	3.37 ± 1.01c	9.21 ± 0.99b	11.01 ± 1.21a
12	Sinapic acid	24.5 ± 0.16	324	nd	0.09 ± 0.02a	0.07 ± 0.02a	0.06 ± 0.01a	0.08 ± 0.01a	0.05 ± 0.01a
13	*t*-Ferulic acid	26.1 ± 0.14	315	nd	nd	nd	nd	nd	nd
14	Naringin	28.4 ± 0.13	285	0.13 ± 0.04c	0.30 ± 0.08b	0.46 ± 0.10a	0.14 ± 0.06c	1.39 ± 0.47a	1.03 ± 0.33b
15	2.3-diMeO benzoic acid	28.7 ± 0.14	299	0.46 ± 0.11c	0.99 ± 0.14b	1.97 ± 0.24a	0.07 ± 0.02b	1.66 ± 0.55a	nd
16	Benzoic acid	29.4 ± 0.14	275	nd	0.65 ± 0.13	nd	0.03 ± 0.01c	0.76 ± 0.11b	1.24 ± 0.41a
17	*o*-Coumaric acid	30.2 ± 0.16	276	0.04 ± 0.01a	0.06 ± 0.02a	nd	nd	0.020 ± 0.008	nd
18	Quercetin	39.9 ± 0.24	367	nd	0.30 ± 0.09a	0.17 ± 0.08b	0.31 ± 0.12c	3.40 ± 0.98a	1.03 ± 0.34b
19	*t*-Cinnamic acid	42.7 ± 0.23	276	nd	nd	nd	nd	nd	nd
20	Naringenin	46.0 ± 0.04	290	0.28 ± 0.10b	0.64 ± 0.12a	0.11 ± 0.03c	0.04 ± 0.01a	0.01 ± 0.004b	nd


*nd: not determined; ^∗^Values expressed are means ± SD; Data marked with different letters within the same row indicate statistically significant differences for each sample (*p* < 0.05).*

### Determination of the Total Phenolics, Flavonoids, Saponins, and Triterpenoids Content

Besides identified compounds, many other compounds might be responsible for the biological effects of investigated species. In this study, the values of total phenolics, flavonoids, saponins, and triterpenoids content are shown in **Table 1**. Total phenolics content of *Potentilla* extracts varied from 24.98 to 138.45 mg GAE/g. Ps-Wat (138.45 mg GAE/g extract) had the highest phenolics content, whereas the Ps-EA (24.98 mg GAE/g extract) had the lowest content. Contrary to our results, Sohretoglu et al. (2015) found that ethyl acetate extract of *P. recta* and *P. astracanica* Jacq. contained higher total phenolics content compared to butanol and water extracts. Tomovic et al. (2015) reported that the total phenolics content was 116.0 mg GAE/g in water extracts of *P. reptans* which is lower than for Pr-Wat (135.73 mg GAE/g), in the present study. The total phenolics content of different solvent extracts of *P. atrosanguinea* was reported by Gupta et al. (2016) who found that the hydroalcoholic extract showed higher phenolic content (429.8 mg GAE/g dry weight of extract). Several authors also reported the total phenolics contents of *Potentilla* species indicating them as valuable sources of bioactive compounds (Tomczyk et al., 2010; Wang et al., 2013; Sohretoglu et al., 2015).

The total flavonoids content of *Potentilla* species ranged from 9.34 to 37.95 mg RE/g extract. The highest values were obtained from Pr-Met, Pr-Wat, and Ps-Met with 37.95, 30.56, and 29.83 mg RE/g extract, respectively. The lowest total content value was obtained from Ps-EA with 9.34 mg RE/g extract. Total flavonoids content of some *Potentilla* species was also reported by Tomczyk et al. (2010), Sohretoglu et al. (2015), and Tomovic et al. (2015). As for total saponins content, Ps-Met (928.05 mg QE/g extract) contained the highest total saponins content, followed by Pr-EA (506.81 mg QE/g) and Pr-Met (459.93 mg QE/g). According to the results, water extracts contained lower total saponins content than other extracts. These findings agree with the previous results obtained by Uysal and Aktumsek (2015). Nonetheless, Ps-Met (5.17 mg OAE/g) had the highest total triterpenoids content. The lowest amount of total triterpenoids content was obtained from Pr-Wat.

### Free Radical Scavenging Activity and Total Antioxidant Capacity

The free radical scavenging activity of *P. reptans* and *P. speciosa* was evaluated using the DPPH and ABTS radical scavenging assays. As shown in **Table 3**, the values of DPPH radical scavenging activity for *P. speciosa* and *P. reptans* ranged from 46.51 to 334.66 mg TE/g and from 0.61 to 4.55 mmol TE/g in ABTS radical scavenging activity, respectively. In the DPPH assay, Pr-Met and Ps-Met displayed more potent radical scavenging activity than other extracts. Additionally, in the ABTS assay, Pr-Wat (4.55 mmol TE/g extract) and Ps-Wat (4.17 mmol TE/g extract) had the highest radical scavenging activity among all samples. Tomovic et al. (2015) reported that the aerial parts and rhizome extracts of *P. reptans* showed DPPH radical scavenging activity with IC_50_ value of 12.11 and 2.57 μg/ml. In another study, Gupta et al. (2016) found that H_2_O/MeOH crude extract of *P. atrosanguinea* exhibited the highest DPPH radical scavenging activity (90.04 %) followed by EtOAc (88.10%) and *n*-BuOH (82.37%) at 200 μg/ml. Besides, Liu et al. (2016) reported that the ABTS values of *P. fruticosa* varied from 303 to 1309 μmol TE/g. In addition, researchers have reported that different *Potentilla* species have important radical scavenging activities (Choudhary et al., 2013; Rauf et al., 2013; Wang et al., 2013).

**Table 3:** Antioxidant properties of different solvent extracts obtained from of *P. reptans* and *P. speciosa^∗^.*

Assays	*Potentilla reptans*			*Potentilla speciosa*		
		
	Ethyl acetate	Methanol	Water	Ethyl acetate	Methanol	Water
DPPH scavenging (mg TEs/g extract)^a^	119.54 ± 4.97c	331.98 ± 1.26a	292.10 ± 6.57b	46.51 ± 6.82c	334.66 ± 0.70a	200.54 ± 9.88b
ABTS scavenging (mmol TEs/g extract)^a^	2.06 ± 0.12c	4.39 ± 0.06b	4.55 ± 0.19a	0.61 ± 0.07c	3.62 ± 0.10b	4.17 ± 0.19a
CUPRAC (mg TEs/g extract)^a^	131.03 ± 2.18b	263.39 ± 3.11a	261.41 ± 1.88a	91.85 ± 1.93b	264.62 ± 3.25a	269.18 ± 3.37a
FRAP (mg TEs/g extract)^a^	81.59 ± 1.76c	204.10 ± 0.39b	219.97 ± 2.06a	56.97 ± 0.82c	191.53 ± 3.47b	214.49 ± 1.84a
Phosphomolybdenum (mmol TEs/g extract)^a^	1.64 ± 0.06c	2.62 ± 0.02b	2.73 ± 0.15a	1.12 ± 0.03c	2.34 ± 0.02b	3.03 ± 0.08a
Metal chelating (mg EDTAEs/g extract)^b^	6.87 ± 0.33c	20.04 ± 0.51b	32.86 ± 0.07a	3.45 ± 0.76c	9.09 ± 1.33b	26.94 ± 1.70a


*^a^TEs, trolox equivalents; ^b^EDTAEs, disodium edetate equivalents; ^∗^Values expressed are means ± SD; Data marked with different letters within the same row indicate statistically significant differences for each sample (*p* < 0.05).*

Total antioxidant capacities of *Potentilla* extracts were determined using phosphomolybdenum assay and the results were depicted in **Table 3**. Ps-Wat, Pr-Wat, and Pr-Met exhibited important total antioxidant capacities with the values of 3.03, 2.73, and 2.62 mmol TE/g extract, respectively. According also to the DPPH and ABTS results, the ethyl acetate extracts of both investigated *Potentilla* species demonstrated lowest antioxidant activity in phosphomolybdenum assay.

### Reducing Power and Metal Chelating Activity

FRAP and CUPRAC assays were used to determine reducing power activity of *Potentilla* species. The water extracts exhibited more pronounced activity as compared to other extracts in both CUPRAC and FRAP assays (**Table 3**). The lowest reducing power activity was observed for ethyl acetate extracts. The reducing power activity of the methanol extract of *P. speciosa* root (FRAP: 133.35 and CUPRAC: 189.24 mg TE/g) was significantly lower than results obtained for aerial parts of *P. speciosa* (FRAP: 191.53 and CUPRAC: 264.62 mg TE/g) (Zengin et al., 2016). Liu et al. (2016) reported that the FRAP values of *P. fruticosa* collected from eight locations ranged from 112.24 to 436.58 μmol TE/g. In addition, Gupta et al. (2016) evaluated CUPRAC activities of different fractions of root extract of *P. atrosanguinea* in which the H_2_O/MeOH extract exhibited the highest reducing activity followed by *n*-BuOH, EtOAc, and H_2_O fraction at 200 μg/ml.

In the metal chelating assay, Pr-Wat (32.86 mg EDTAE/g) and Ps-Wat (26.94 mg EDTAE/g) were the most active, whereas the Pr-EA (6.87 mg EDTAE/g) and Ps-EA (3.45 mg EDTAE/g) were the least active. The ethyl acetate extracts of the studied *Potentilla* extracts showed the lowest metal chelating activities and these results are supported as well by the previous findings of Uysal and Aktumsek (2015). In our previous study, we reported that metal chelating activity for the methanol extract of *P. speciosa* root was 4.32 mg EDTAE/g (Zengin et al., 2016), and this value is lower than the one obtained herein for aerial part of *P. speciosa* (9.09 mg EDTAE/g).

### Enzyme Inhibitory Activity

The inhibitory activities of tested extracts against cholinesterases (AChE and BChE), α-amylase, α-glucosidase, and tyrosinase were tested and the results are presented in **Table 4**. The inhibitory activities against all enzymes ranged according to the extraction solvents. Generally, the water extract demonstrated a lower activity against all enzymes. The ethyl acetate and methanol extracts showed prominent inhibitory effects against AChE. Moreover, the highest BChE inhibitory activities were obtained from the Pr-EA and Ps-Wat with 6.15 and 2.87 mg GALAE/g. However, Ps-Met was inactive against BChE. There are several reports in literature indicating that terpenoid and phenolic compounds have promising cholinesterase inhibitory activities (Orhan et al., 2007; Stasiuk et al., 2008; Bahadori et al., 2016). Accordingly, terpenoid and phenolic rich *Potentilla* species could be considered as promising AChE and BChE inhibitors.

**Table 4:** Enzyme inhibitory activities of different solvent extracts obtained from of *P. reptans* and *P. speciosa^∗^.*

Assays	*Potentilla reptans*			*Potentilla speciosa*		
		
	Ethyl acetate	Methanol	Water	Ethyl acetate	Methanol	Water
AChE Inhibition (mg GALAE/g extract)^a^	3.99 ± 0.08a	3.56 ± 0.22b	1.30 ± 0.22c	3.55 ± 0.22b	3.76 ± 0.06a	0.71 ± 0.06c
BChE Inhibition (mg GALAE/g extract)^a^	6.15 ± 0.37a	0.09 ± 0.01c	0.52 ± 0.01b	1.43 ± 1.17c	nd	2.87 ± 0.27a
α-Amylase inhibition (mmol ACE/g extract)^b^	1.99 ± 0.07a	1.29 ± 0.18b	0.36 ± 0.01c	1.46 ± 0.25b	2.41 ± 0.44a	0.38 ± 0.01c
α-Glucosidase inhibition (mmol ACE/g extract)^b^	4.94 ± 1.70c	54.19 ± 0.57a	40.99 ± 2.62b	2.80 ± 0.93c	54.57 ± 0.14a	38.59 ± 6.20b
Tyrosinase inhibition (mg KAE/g extract)	108.56 ± 6.13b	123.36 ± 4.63a	31.48 ± 4.19c	106.54 ± 9.63b	144.39 ± 1.49a	35.60 ± 4.12c


*^a^GALAEs, galantamine equivalents; ^b^ACEs, acarbose equivalents; ^c^KAEs, kojic acid equivalents nd: not determined. ^∗^Values expressed are means ± SD. Data marked with different letters within the same row indicate statistically significant differences for each sample (*p* < 0.05).*

The anti-diabetic activity of *Potentilla* species was investigated by testing their inhibition abilities on α-amylase and α-glucosidase (**Table 4**). In α-glucosidase inhibitory activity, Pr-Met and Ps-Met showed the highest inhibitory activities with the values of 54.19 and 54.57 mmol ACE/g, respectively. Similarly, to our results, Kumar et al. (2013) reported that methanol extracts from *P. fulgens* have strong inhibitory activity against α-glucosidase, and ethyl acetate fraction exhibited potent α-glucosidase inhibitory activity. In addition, these authors found that isolated terpenoids from *P. fulgens* demonstrated significant α-glucosidase inhibitory activity. Similarly, several triterpenoids and phenolics exhibited antidiabetic activities (Ivorra et al., 1988; Li et al., 2008; Luo et al., 2008; Etxeberria et al., 2012; Ali Asgar, 2013). Thus, the presence of these components in *Potentilla* species could be correlated with the observed antidiabetic activity.

The tyrosinase inhibitory activity of the studied *Potentilla* species ranged from 31.48 to 144.39 mg KAE/g. As shown in **Table 4**, Ps-Met (144.39 mg KAE/g) and Pr-Met (123.36 mg KAE/g) displayed remarkable tyrosinase inhibitory activities. Additionally, the lowest tyrosinase inhibitory activities were observed in Pr-Wat (31.48 mg KAE/g) and Ps-Wat (35.60 mg KAE/g).

### Cytotoxicity

Although some of the best anticancer drugs are from natural origin or derived, they present also negative effects on human health. Therefore, investigation of medicinal plants for discovery of potent anticancer compounds having fewer side effects is warranted. In this work, the cytotoxic activity of extracts from *P. reptans* and *P. speciosa* was determined against two human cancer cell lines (A549 and MCF-7). IC_50_ values were expressed as mean of quadruplicates ± SD (**Table 5**). The highest cytotoxicity was observed for water extract of *P. reptans* (IC_50_ < 130 μg/ml). Compared to the positive control, doxorubicin (IC_50_ = 9.6 and 2.5 μg/ml against A549 and MCF-7 cells in 48 h, respectively), *P. reptans* showed high antiproliferative activity against MCF-7 cells. *P. speciosa* exhibited weak to moderate activity against both of A549 and MCF-7 cell lines. The cytotoxicity rate of A549 and MCF-7 cells was found to be time dependent (**Table 5**). In general, crude extracts with IC_50_ values less than 1000 μg/ml could be considered to be active. Several classes of natural compounds found in *Potentilla* species could be responsible for their antiproliferative activity. Previous studies revealed that triterpenoids, tannins and phenolic compounds isolated from *Potentilla* species exhibited cytotoxic activities against some human cancer cell lines (Li et al., 2007; Tomczyk and Latté, 2009; Choudhary et al., 2013; Rauf et al., 2015). For example two flavonoids (such as chrysin) from *P. evestita* Th.Wolf showed prominent cytotoxic and antitumor promoting properties (Rauf et al., 2015). Also, DNA topoisomerase I and II inhibitory activity has been observed for phenolic compounds isolated from *P. argentea* L. (Tomczyk et al., 2008). In comparison to previous studies, *P. reptans* exhibited moderate to high cytotoxicity. According to our literature review, this is the first report concerning the antiproliferative activity of extracts obtained from *P. reptans and P. speciosa*. However, further phytochemical and pharmacological studies are needed for identification of responsible compounds and evaluation of the molecular mechanism of their anticancer action.

**Table 5:** Cytotoxicity of different solvent extracts obtained from *P. reptans* and *P. speciosa* (IC50 μg/ml)*^∗^*.

Cell line	Time (h)	*Potentilla reptans*			*Potentilla speciosa*			Doxorubicin
				
		Ethyl acetate	Methanol	Water	Ethyl acetate	Methanol	Water	
MCF-7	24	156 ± 6.2a	90 ± 2.2b	130 ± 2.5c	482 ± 14*d*	660 ± 35*e*	710 ± 46*e*	7.8 ± 0.4*f*
	48	85 ± 2.8a	55 ± 1.4b	90 ± 3.2a	287 ± 15c	390 ± 18*d*	425 ± 16*e*	2.5 ± 0.1*f*
	72	65 ± 1.4a	42 ± 1.2b	70 ± 2.2a	248 ± 6.4c	312 ± 7.5*d*	350 ± 11*e*	2.1 ± 0.1*f*
A549	24	490 ± 9.0a	443 ± 8.4b	89 ± 1.6c	101 ± 2.5*d*	990 ± 38*e*	705 ± 22*f*	22.4 ± 1.1*g*
	48	298 ± 8.1a	305 ± 10a	65.5 ± 3.3b	73.6 ± 4.9b	662 ± 19c	430 ± 11*d*	9.6 ± 0.6*e*
	72	163 ± 3.5a	180 ± 4.4b	52 ± 1.2c	60 ± 1.4*d*	424 ± 8.8*e*	297 ± 10*f*	8.2 ± 0.7*g*


*^∗^Values expressed are means ± SD. Data marked with different letters indicate significant difference (*p* < 0.05).*

## Conclusion

To sum up all, in the present work, different biological effects for two *Potentilla* species were observed as well as their chemical profiles. From our results, it was apparent that the biological activities and chemical profiles were dependent on extraction solvents and their polarity. Rutin and catechin were the major phenolic components identified in these extracts. Generally, the methanol and water extracts showed higher antioxidant activities as compared to ethyl acetate extracts. Furthermore, investigated *Potentilla* species revealed good inhibitory properties on tested enzymes linked to major health problems (AD and DM), and MCF-7 cells. From the present results, these two *Potentilla* species could be considered as promising sources of natural-biologically active agents for pharmaceutical and food industries. However, further experimental studies such as *in vivo* animal models and toxicological assays are recommended for the studied *Potentilla* species.

## Author Contributions

SU, GZ, ML, MB, AnM, GB, and ED set up and carried out experiments. AdM and AA executed data analysis.

## Conflict of Interest Statement

The authors declare that the research was conducted in the absence of any commercial or financial relationships that could be construed as a potential conflict of interest.
